# Implementing a digital platform for the oncology unit at Dalal Jamm hospital, Senegal

**DOI:** 10.3389/fdgth.2026.1750131

**Published:** 2026-07-29

**Authors:** Coumba Gueye, Amacoumba Fall, Jafaar Thiam, Gorgui Sarr, Sidy Ka, Salif Balde, Mariéme Guetti

**Affiliations:** 1 Centre Hospitalier National Dalal Jamm, Dakar, Senegal; 2 Faculty of Medicine and Pharmacy, Cheikh Anta Diop University, Dakar, Senegal; 3 Oncology Department at Centre Hospitalier National Dalal Jamm, Dakar, Senegal

**Keywords:** digital health platform, implementation evaluation, oncology, quality care, Senegal

## Introduction

According to statistics, the annual incidence of cancer in Senegal is estimated at 6,646 new cases, with a mortality rate of approximately 72%. Breast and cervical cancers are the most prevalent types (1). The majority of patients receiving cancer care are referred to Dalal Jamm Teaching Hospital, which is committed to providing a high standard of care, including palliative care services.

Although the expertise needed for adequate care exists within the center, some challenges remain regarding patient needs, prescriptions, and follow-ups. Patient records—including updates, prescriptions, and test results—are still paper-based, with single-handed reports shared between clinicians. Data errors are fairly common (2). In addition, data loss can lead to delayed decisions and barriers to quality care. In the United States, a study conducted in the emergency department stated that small data errors affected the ability to determine the department’s operational performance (3). Some studies conducted in developing countries concluded that Electronic Health Records (HER)helped health professionals reduce medical errors, achieving better care coordination and improving safety and quality (4, 5).

Following these inquiries, we harmonized an implementation strategy based on the following question: How can local issues be overcome given existing resource constraints? EHRs represent digital versions of a patient's record of care. Accessible to all clinicians involved, the information can be updated and shared within the community health system for timely decision-making (6).

Moreover, a digital platform with coded access will ensure data collection, quick access to records, and traceability of inputs (7).

With wearable devices, mobile applications, telemedicine, and Artificial Intelligence, EHRs represent an important component of digital health technology (8, 9).

As a new concept, understanding the process and adopting appropriate materials to support the implementation effort are essential.

According to the World Health Organization, digital health literacy represents the ability to seek, find, understand, and appraise health information from electronic sources and to apply that knowledge to solve health-related problems (10).

The WHO published the Digital Health Platform Handbook (DHPH), a toolkit aimed at helping countries digitalize their healthcare system.

Globally, teams have developed platforms enabling direct access to clinical data, patient outcome monitoring, data extraction, and improved quality of care and service delivery (11–16).

However, in the Senegalese context, data are still collected on paper.

To address this gap, we aimed to develop a dynamic platform—Papyrus—for centralized data, where the attending physician can edit patient medical information, adding inputs, test results, radiology images, histopathology results, expert notes, allergy status, medication records, and discharge alerts.

The first report will seek to assess the availability of a functional, adopted digital platform in oncology, which is a meaningful and necessary milestone for enabling future patient and population impact. Moreover, adoption, reach, and feasibility will be reported.

## Methodology

The implementation support required specific materials, connectivity, and competencies.

First, we assessed the needs and assets of the department of oncology based on organizational, clinical, and usual investigations.

The developer required computers with the following characteristics:

Computer fix hp Processor Intel Core i7.
16Go memory vive DDR4 2666 MHz 1TB.

Once the needs were identified, we were able to design areas for improvement and plan the evaluation model. Consequently, the platform and evaluation plan were drafted.

The IT department was already providing internet access for the administrative office. However, the program needed additional connection for the outpatient unit, dedicated room for data storage with adequate software, and one IT specialist for management and maintenance.

Then, the experts needed for holistic care (one radiation oncologist, five surgeons, two medical oncologists, one palliative care worker, one social worker, two nurses, one navigator, and an archivist) and the developer were invited for the project presentation. Later, we organized a one-day workshop in order to determine the different items and their translation into tabs on the platform.

To ensure continuity, a session for validation was organized.

Lastly, three rounds of training were organized between the developer and the providers before the first week of utilization of the platform.

The training was initially held by the developer and aimed to improve the capacity of entering data, uploading results, and secure usability of the platform with 35 participants. Two residents who manage basic software issues performed the second and third sessions, attended by 40 participants.

The program's effectiveness was assessed through Quality Assurance and Performance Improvement protocols. Key parameters included system access, user interface functionality, connectivity, and the flow of information across departments—including radiation oncology, surgery, medical oncology, inpatient and outpatient services, and palliative care—as a determining factor.

## Results

From June 2024 to September 2025, no chart loss was noticed.

Instant access to medical records with a high flow of information between providers was reported.

**Figure 1:**
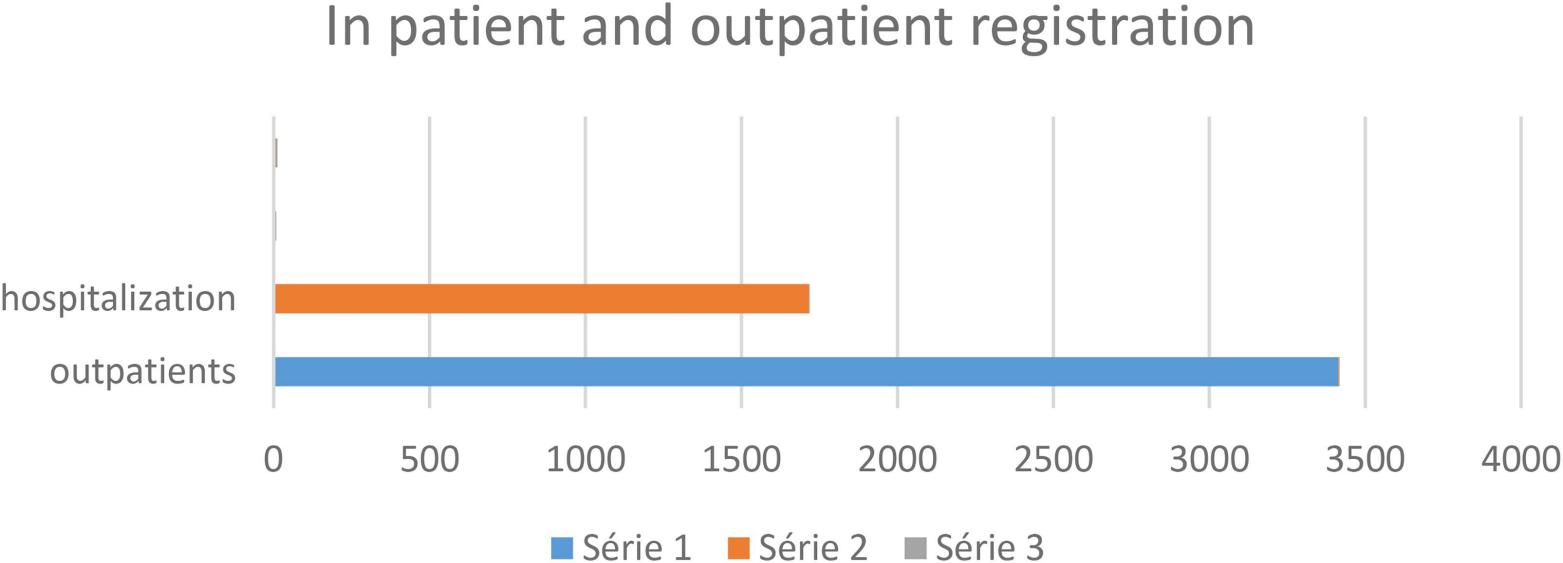
Number of patients registered.

**Figure 2:**
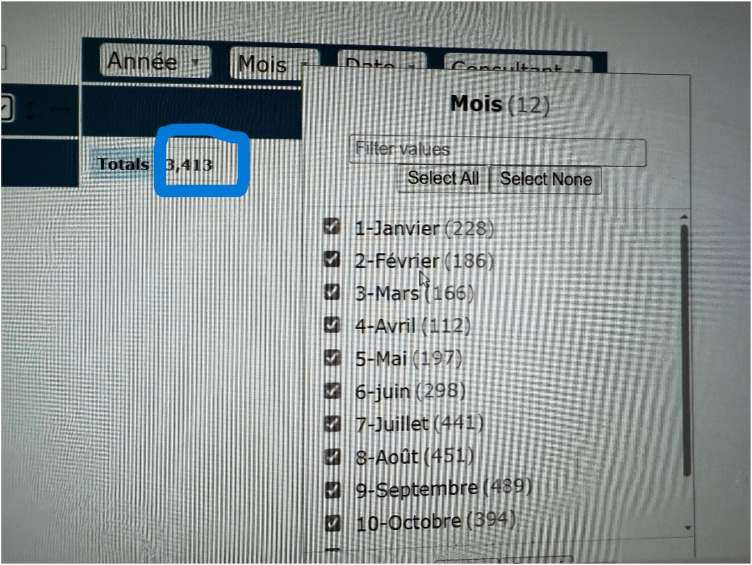
Number outpatient registration.

The number of outpatients and inpatients registered were, respectively, 3,413 and 1,717 (Figures 1, 2).

**Figure 3:**
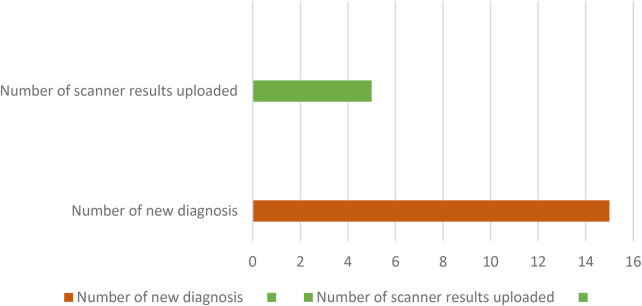
New diagnoses.

The group of individuals newly diagnosed with cancer was 105 but only five scanner results were uploaded (Figure 3). Considering that two scanners were available in one outpatient room for examinations, one room for chemotherapy follow ups, and one office for inpatient admission, this low rate was not expected. However, the feedback for the first week of activity stated that only the scanner in the outpatient was used to upload radiology images.

**Figure 4:**
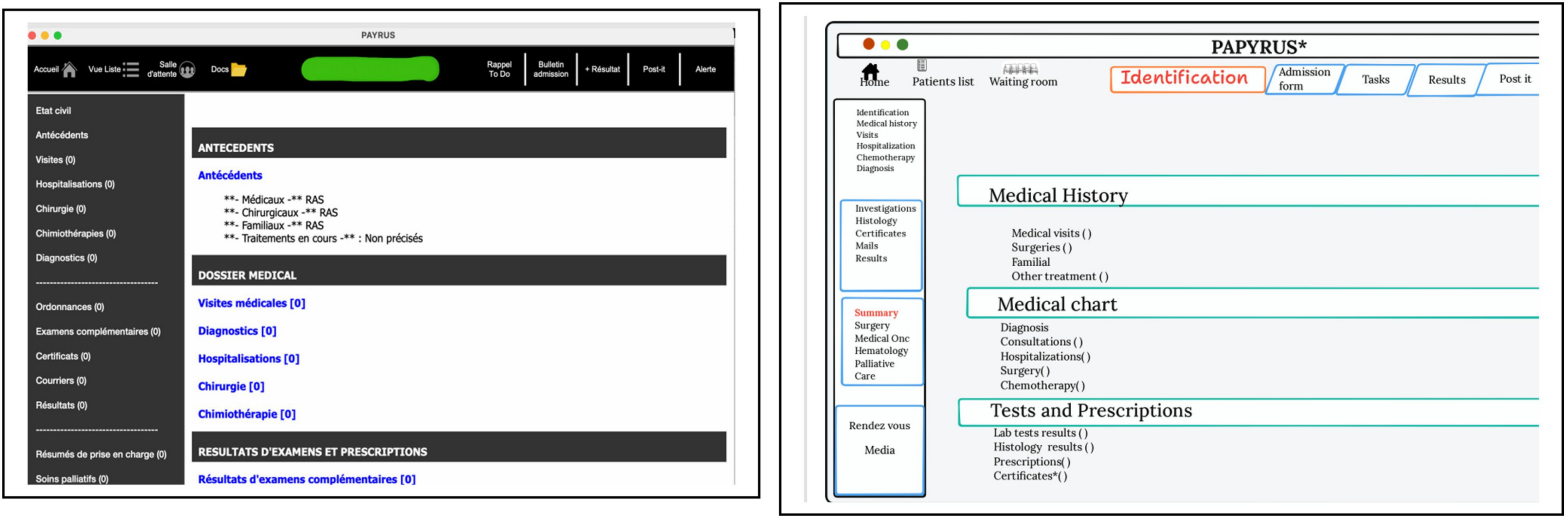
Home page with summary tab.

Moreover, the patient’ summary, which was automatically generated and available under one specific tab, represented an innovative parameter and was highly useful for follow ups (Figure 4).

It was also possible to measure the length of a patient’s stay in the outpatient unit and to trace referrals.

## Discussion

Developing a platform for medical records was highly needed in the Senegalese context. The project aimed to develop a dynamic system for centralized data. Moreover, the system offered an opportunity for attending physicians to edit patient’s medical information, insert diagnosis details, share decisions, and insert alerts.

Reviewing the literature, different studies emphasized the benefits of EHR on quality of care through medical practice (15, 17–21).

In our institution, the most significant advantage reported by the team was direct access to the patient’s records. That opportunity was in fact also a motivation for the adoption of the project. However, the implementation team stated platform access being limited to the hospital setting was an obstacle. In this regard, the project conforms to the national guidelines about personal data management. Legally, data collected in the country must be stored in the country, except for special authorization from accredited authorities.

As highlighted in Figure 3, we noticed a limited number of radiographic images uploaded. The feedback received highlighted issues involved with residents who were trained in rotation at other institutions and those who were absent during training sessions.

For the first week, the limited number of scanner machines available represented a barrier to uploading more images. Over the following weeks, the inability to upload images to the platform forced users to manually record their results in charts.

Based on previous studies, improving digital health literacy for professionals is crucial for person-centered care (22–24).

In order to improve that parameter, we need to repeat required training about EHR navigation and digital health literacy. For adoption and sustainability of the platform, the assessment of needs and feedback collection represent a continuous activity to improve staff competencies and EHR usability.

As summarized below (Box 1), three themes will be considered for a better outcome:

BOX 1 **Core factors for impact on implementation adoption and outcome.**

**Table:** 

1. Training	2. Acceptance	3. Workflow
Initial: improve capacity and secure usability of the platform	Triggered by enabling direct access to charts	Redefine the system selection implementation process
Ongoing with two residents who manage basic software issues	Sustained by the opportunity for data extraction for research	Adaptations with needs identified

Furthermore, the impact on patient outcomes will be evaluated in the long-term report, in accordance with the QAPI plan.

## Conclusion

Medical digitalization is an innovative concept in the Senegalese context. With the platform, data collected is secure and accessible and decisions are traceable. The system requires continuous evaluation to improve efficiency and user competence.

Aligning with legal national guidelines, the team will seek authorization for online use.

## Data Availability

The raw data supporting the conclusions of this article will be made available by the authors without undue reservation.
